# The Action of Drugs on the Kidney

**Published:** 1888-01

**Authors:** 


### THE ACTION OF DRUGS ON THE KIDNEY.

The action of drugs on the circulation and secretion of the kidney is a subject as interesting as it is comparatively little known. A brief and valuable contribution to the subject was made by Dr. C. D. F. Phillips, of London, to the Ninth International Medical Congress, and published in the *Lancet* of November 12, 1887. The experiments were made on cats and dogs. The results arrived at may be summarized as follows:

The effects of caffeine citrate, in half-grain doses: The *blood-pressure* is first lowered, and is then raised, both effects being of short duration and slight, especially the rise of pressure. The *heart* showed first a diminution in the force of the beats, followed by a slowing, with beats of markedly increased force. On the *kidney* caffeine causes at first contraction, lasting two or three minutes, and accompanied by diminution or even arrest of secretion; this is followed by expansion, that lasts, after a one-grain dose, more than half an hour sometimes, the flow of urine being at the same time trebled. That the effects on the kidney cannot be due to the changes in the general blood-pressure, is shown by the fact that they do not occur simultaneously.

The effects on the general blood-pressure are slight, and last only a few seconds, the effects on the kidneys being measured by minutes.

Ulexine, the new alkaloid obtained from the seeds of the common gorse, acts in a similar way to caffeine. The objection to it is that its diuretic action is maintained only by doses that would either kill or produce violent convulsions. Like caffeine, it produces first a constriction and then an expansion of the renal vessels, with diuresis. It differs from caffeine, 1, in being more powerful; 2, its effects are more transitory; 3, repeated doses of caffeine injected rapidly cause only contraction of the kidney, not followed by expansion. A similar excess of ulexine causes expansion only, without contraction. Other substances that cause expansion of the kidney are dextrose, urea, acetate and chloride of sodium, and probably all constituents of the urine. More numerous are the drugs causing constriction of the kidney. Digitalin causes contraction in doses of $\frac{1}{40}$ gr., which persists for as much as half an hour. While it is difficult to say if digitalis is a true diuretic, or only exerts its effects through the heart, the urine is not diminished during the contraction of the kidney, as is the case with caffeine, but it is generally increased. The explanation probably lies in the effect on the general blood-pressure, digitalin raising and caffeine lowering it.

While spartein acts similarly to digitalin on the heart, general blood-pressure, and renal vessels, it causes a great diminution of urine; and its so-called diuretic effect, in disease, is due to improvement of the general circulation. The chief action of strophanthin and apocynëin is on the heart muscle, and they produce little or no effect on the peripheral vessels. Turpentine, adonidin and barium chloride produce marked renal contraction without diuresis. From the above it is seen that "reputed diuretics more commonly produce contraction of the renal vessels than expansion. Further, that expansion is either slight, as by acetate of soda; or, if large, as by citrate of caffeine, it is only produced by small and initial doses. The powerful action of alexin on the respiratory mechanism is a great drawback to its use; one-sixth of a grain was used in our experiments, but one-twelfth of a grain would completely arrest respiration. Then, again, such drugs as produce contraction of the kidney cannot be bracketed together, since, though they all have the same effect on blood-pressure, digitalin alone has an obviously diuretic effect." The flow of urine is not so much dependent on blood-pressure as on the rate of the blood-flow in the renal vessels. "It is necessary also to remember that although such drugs as strophanthin produce a great increase in the force of the cardiac beats, yet the heart is very much slowed, so that it is possible that the amount of blood sent into such an organ as the kidney in a given time may remain the same; whereas, such a drug as digitalis, producing a rise of blood-pressure and a contraction of the kidney vessels, may cause an increased quantity of blood to pass through those vessels, and thus it acts as a diuretic. Inasmuch as spartein has not a marked diuretic action, we must also assume that digitalin has some peripheral action on the secretory apparatus of the kidney." Dr. Phillips concludes his paper by calling attention to the value of the plethysmographic method of experimentation in regard to the action of drugs on the circulation.—*Journal of The American Medical Association.*

